# Conceptual Closure Elicited by Event Boundary Transitions Affects Commercial Communication Effectiveness

**DOI:** 10.3389/fnins.2020.00292

**Published:** 2020-04-07

**Authors:** Richard Silberstein, Shaun Seixas, Geoffrey Nield

**Affiliations:** ^1^Centre for Human Psychopharmacology, Swinburne University of Technology, Hawthorn, VIC, Australia; ^2^Neuro-Insight Pty Ltd., Hawthorn, VIC, Australia

**Keywords:** event boundary, long-term memory encoding, steady state visual evoked potential, prefrontal cortex, conceptual closure

## Abstract

While our experience of the world may appear continuous, recent evidence suggests that our experience is automatically segmented and encoded into long-term memory as a set of discrete events. Event segmentation is an important process in long-term memory encoding with evidence pointing to experiences occurring around event boundaries being better recognized subsequently. Neuroimaging studies have shown increased activity in the hippocampus and other nodes of the default mode network (DMN) when encountering an event boundary. We have previously demonstrated that the steady state topography (SST) measure of brain activity at a left inferior frontal scalp sites is correlated with the strength of long-term memory encoding. More recently, we have noted that event boundaries occurring in naturalistic stimuli such as television advertising trigger a transient drop in activity at the inferior frontal scalp sites, an effect we have termed *Conceptual Closure*. In this study, SST measures of brain activity were recorded in 50 male participants as they viewed a first-person journey through a 10-room virtual art gallery. We hypothesized that the transition from one room to another would serve as an event boundary which would triggers increased hippocampal and DMN activity while correspondingly decreasing activity in task positive networks in the vicinity of the inferior frontal cortex thus eliciting Conceptual Closure. A permutation test confirmed the hypothesis in that the appearance of the door between gallery rooms was associated with Conceptual Closure in that we observed a significant drop in brain activity at the left hemisphere inferior frontal scalp site at this point in time. Finally, we illustrate the real-world impact of Conceptual Closure by considering the commercial effectiveness of a television advertisement that exhibited Conceptual Closure at points of branding. The television advertisement was broadcast before and after it was re-edited to minimize Conceptual Closure at the time the advertising brand was being featured. Minimizing Conceptual Closure at the time of branding and key message was associated with significant increased commercial effectiveness of the advertisement.

## Introduction

While our experience of the world may appear continuous, recent evidence suggests that our experience is segmented and encoded into long-term memory as a set of discrete events (Zacks and Swallow, 2007; Kurby and Zacks, 2008). Such segmentation appears to be automatic and operating on multiple timescales with finer temporal components constituting subcomponents of larger scale segments (Swallow et al., 2009). In the current study, we examine the impact of event boundaries on long-term memory encoding. The motivation for this study emerged from our observation of the brain activity correlates of long-term memory encoding when viewing naturalistic time extended stimuli such as television advertisements or television programs. We found that event boundaries in the viewed material were frequently associated with transient drops in long-term memory encoding and the current study aims to examine this effect using a naturalistic stimulus with repeated, well-defined and consistent event boundaries. We conclude our report with an illustrative example of the potential impact of such event boundaries on the commercial effectiveness of a television advertisement.

Evidence for segmentation of experience was originally suggested by the inter-subject consistency of the location of event boundaries in video content (Newtson, 1973; Newtson et al., 1977; Speer et al., 2003). Event segmentation also appears to play a role in long-term memory encoding with evidence pointing to material occurring around an event boundary being better recognized subsequently (Swallow et al., 2009). While events occurring around an event boundary appear to be better recognized, there is evidence that working memory is updated at an event boundary. For example, participants walking through a doorway in a virtual reality experience exhibit reduced recall for content and events prior to walking through the doorway, presumably an event boundary (Radvansky and Copeland, 2006).

Neuroimaging studies also indicated changes in brain activity when encountering an event boundary. For example, higher levels of brain activity were observed when encountering an event boundary in either film or reading (Kurby and Zacks, 2008; Zacks et al., 2010; Ben-Yakov et al., 2013). Furthermore, while increases in brain activity have been observed to coincide with the occurrence of consciously identified event boundaries, there may be other events that are processed as event boundaries but are not consciously recognized as such. Nakano et al. (2013), Nakano (2015) examined the brain activity correlates of blinks. They found that blinks tend to occur at what the authors describe as ‘breakpoints’ or points in time coinciding with event boundaries such as a pause in speaking or the end of a sentence while reading (Nakano et al., 2013). The blink related changes in brain activity reported by Nakano et al. (2013), Nakano (2015) reveal richer detail in the changes in brain activity associated with blink related event boundaries. Specifically, brain activity increases immediately following blinks were primarily restricted to the default mode network (DMN), including the hippocampus while activity in the dorsal attention network, a task positive network, was reduced (Nakano et al., 2013, Nakano, 2015). This was interpreted as a transient disengagement from sensory or external perception and the engagement of *internally oriented* networks (Nakano, 2015).

Earlier observations of enhanced long-term memory encoding for events occurring around the times of event boundaries led researchers to examine the relationship between hippocampal activity and event boundaries (Ben-Yakov et al., 2013, 2014; Ben-Yakov and Henson, 2018). In the most recent study (Ben-Yakov and Henson, 2018), brain activity was measured while participants viewed either an 8.5 min unfamiliar film or a 120 min film they had first viewed some years previously. Five independent observers identified the occurrence of event boundaries in the films and the *event boundary strength* of an event boundary was defined as the number of observers (more than one) that identified the specific event boundary. The findings were consistent with the stated hypothesis in that only hippocampal activity increased monotonically with *event boundary strength*. Furthermore, a data-driven approach indicated that the times of hippocampal activity peaks were associated with the occurrence of event boundaries.

While much of the research concerning the processing of event boundaries have focused on memory processes and especially hippocampal activity, a recent study has revealed a temporal hierarchy of event boundary processing in a number of cortical regions. Using a data driven approach, researchers observed transitions in stable patterns of brain activity as participants viewed an episode of a television serial (Baldassano et al., 2017). These transitions formed a temporal nested hierarchy with the most rapid transitions occurring in the primary visual and auditory cortices, longer transitions occurring in the multimodal cortices and the longest transitions observed in the hippocampus. The longest hippocampal transitions also coincided with human labeled event boundaries.

In summary, both behavioral and brain imaging studies of event boundaries processing suggests that event boundaries trigger transitions in brain activity in a range of cortical regions that occur on multiple time scales and form a nested temporal hierarchy with DMN and hippocampal transitions occurring on the longest time scale. At the level of the DMN and hippocampus, event boundaries trigger subsequent increased activity and a simultaneous reduction in task positive networks such as the dorsal attentional network.

Along with the hippocampus, the ventro-lateral prefrontal cortex (vlPFC) is also known to play an important role in long-term memory encoding. During a memory task, targets associated with higher levels of activity at the vlPFC are better recognized subsequently, an effect known as the ‘differential neural activity based on memory’ and referred to as *Dm* (Buckner et al., 1999; Paller and Wagner, 2002; Blumenfeld and Ranganath, 2007). We have previously demonstrated the *Dm* effect at an inferior frontal site using steady state topography (SST) an evoked potential methodology (Silberstein et al., 2000a). The SST methodology makes use of the steady state visual evoked potential (SSVEP) which is elicited by a diffuse peripheral 13 Hz sinusoidal visual flicker presented at the same time that participants view centrally presented video content The SSVEP phase variations in turn indicate SSVEP latency changes that we interpret in terms of regional brain activity changes with SSVEP latency decreases or *phase advance* indicating increased activity and vice versa (Silberstein et al., 2000b; Kemp et al., 2004). Depending on the context and ease of understanding, we will use the terms *SSVEP latency* and *activity* interchangeably (Silberstein, 1995; Silberstein et al., 2016, 2017, 2019).

Our choice of the SST methodology for this study was based on a number of factors. Firstly, unlike transient evoked potentials such as N200 or P3b, SST is well suited to the study of time extended cognitive tasks such as the ones described in the current study. In addition, the SST methodology is highly resistant to the common sources of EEG artifacts such as muscle activity, blinks and eye movements (Silberstein, 1995; Gray et al., 2003). Finally, the SST measures of brain activity appear to be suitably sensitive to cognitive processes such as visual attention and long-term memory encoding (Silberstein et al., 1990, 2000a,b; Silberstein and Nield, 2008).

In the above mentioned *Dm* study (Silberstein et al., 2000a), we measured variations in 13 Hz SSVEP phase while 35 participants viewed naturalistic television content including unfamiliar advertisements sourced overseas. Advertising images coinciding with SSVEP latency minima or peaks in activity at the left hemisphere lateral frontal scalp site FC5 located between C3 and F7 (overlaying the vlPFC) were better recognized 7 days later than advertising images coinciding with times of minimum activity at this site (66.0% vs. 37.9%, d.f. = 34, *t* = 7.1, *p* < 5 × 10^–8^). As hypothesized, the *Dm* effect was largest at FC5 and the correlation between the 7-day recognition score and the SST measure of activity was only statistically significant at FC5 after correcting for multiple comparisons (Silberstein et al., 2000a).

In the current study, we use SST to examine variations in brain activity while participants encountered event boundaries in a virtual first-person journey through a 10-room art gallery. Each of the rooms displays three paintings that were always viewed at the same pace and followed by an opening door leading to the next gallery room. We hypothesize that the first-person journey through the open door to the next gallery room constitutes an event boundary and that such event boundary will be associated with a transient reduction in activity at FC5, the left hemisphere site we have previously shown to demonstrate a robust *Dm* effect. Our choice of the doors separating the gallery rooms as the event boundaries was based not only on the fact that the doors were the actual boundaries between participants viewing a different trio of paintings but also published reports that passing through doorways triggered reductions in access to memory of items in the preceding room (Radvansky and Copeland, 2006; Radvansky et al., 2011). These findings suggest that doorway passages are processed as event boundaries. In addition, we base our hypothesis of a transient reduction in activity at this site on accumulated evidence that the left and right vlPFCs are nodes of task positive cortical networks. Specifically, the right vlPFC is considered a node of the ventral attentional network (Vossel et al., 2014) while the left vlPFC plays an important role in speech as well as the cognitive control of memory processes (Badre and Wagner, 2007; Blumenfeld and Ranganath, 2007). As both left and right vlPFCs are components of task positive networks, the widely observed negative correlation between DMN activity and task positive networks (Buckner et al., 2008) suggests that the increases in DMN activity associated with the experience of event boundaries should be associated with a transient reduction in activity at the left hemisphere site FC5.

## Materials and Methods

### Participants

The sample comprised 50 male participants, mean age 34.1 years (SD = 12.6 years, range 18–59 years). All were screened for the presence of pre-existing medical, neurological or psychiatric conditions, including epilepsy. Participants were recruited via a recruitment agency and all testing was conducted on the premises of Neuro-Insight Pty Ltd. The study was approved by the Swinburne University Human Research Ethics Committee as application SUHREC2014/029.

### Procedure

**FIGURE 1:**
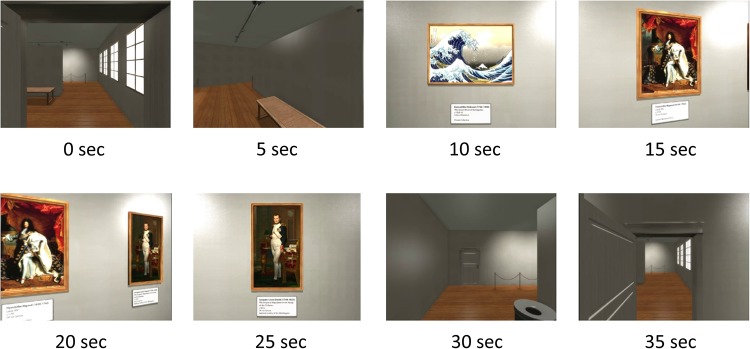
The timing of events in a room in the virtual gallery are illustrated. During the 35 s period in each room, the exit door comes into view at the 30 s mark while the passage through the doorway to the next room occurs at the 35 s mark.

Participants viewed a first-person journey through a 10-room virtual art gallery that was created by the multi-media software company *Opaque Media*. Each room of the gallery exhibited three well known paintings and was entered through a hinged door that opened on approach. Participants spent 35 s in each of the 10 rooms and the entire viewing period occupied 380 s. The timing of the appearance of each painting as well as the appearance of the door to the next room after entering a room was identical for all of the 10 rooms. Views of one of the gallery rooms in 5 s increments are illustrated in Figure 1.

The task was presented on a video monitor subtending 24° vertically and 46° horizontally. The stimulus used to evoke the steady state visually evoked potential (SSVEP) was a white spatially diffuse 13-Hz sinusoidal flicker with a modulation depth of 45% and subtending a horizontal angle of 160° and a vertical angle of 90°, which was superimposed on the visual fields. This flicker was present throughout the task and special goggles enabled subjects to simultaneously view the cognitive task and the sinusoidal flicker.

### Data Acquisition

**FIGURE 2:**
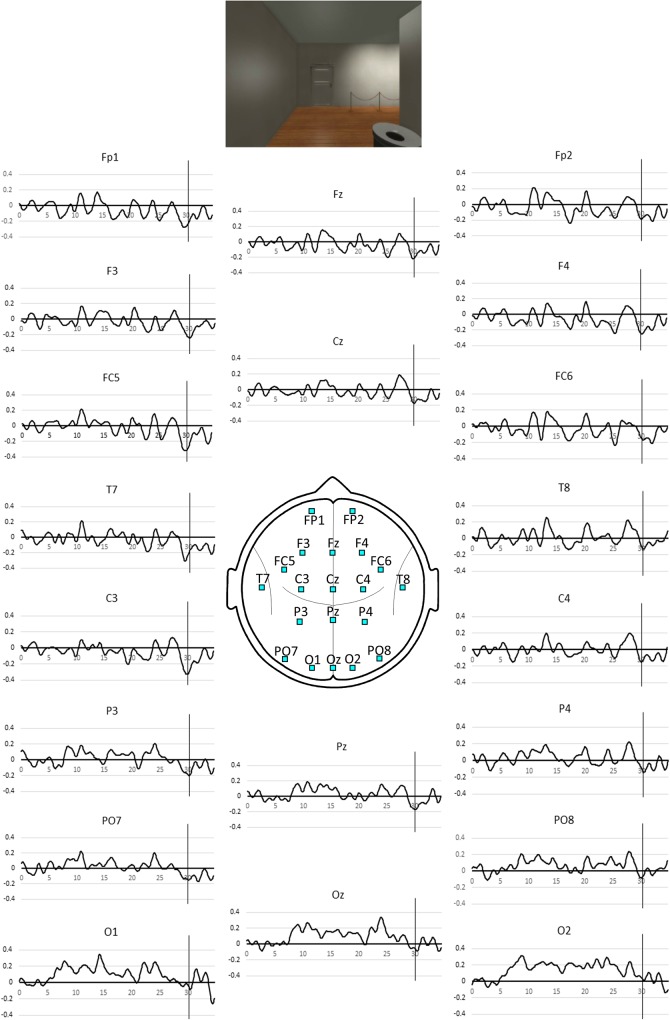
Pooled SSVEP phase averaged over the all gallery rooms for the 35 s period spent in each room. Positive values indicate *phase advance* or increased activity and negative values, *phase lag* or reduced activity. The vertical line indicates the 30 s mark coinciding with the appearance of the exit door as illustrated above. The associated *p*-values for the NULL hypothesis that the observed SSVEP phase at the 30 s mark is not less than the mean value during the first 5 s in the room are listed in Table 1.

Brain electrical activity was recorded from 20 scalp sites illustrated in Figure 2 and these were positioned and labeled according to the International 10-10 system (Acharya et al., 2016). Electrode locations FC5 and FC6 are located closest to the left and right VLPFC respectively. The average potential of both mastoids served as a reference while an electrode located at FPz served as a ground. Brain electrical activity was amplified and bandpass filtered (3 dB down at 0.1 Hz and 30 Hz) before digitization to 16-bit accuracy at a rate of 400 Hz. The major features of the signal processing have been described (Silberstein et al., 2016). Briefly, the SSVEP was determined from the 13-Hz single-cycle cosine and sine (or real and imaginary) Fourier coefficients that were then smoothed using a 10 stimulus-cycle cosine weighted smoothing window. At the stimulus frequency of 13 Hz, this yields a temporal resolution of 380 ms or half the 10-cycle window width because of the cosine weighting. The cosine smoothing window was then shifted 1 stimulus cycle and the coefficients were recalculated for this overlapping period. This process was continued until the entire 380 s of activity was analyzed. An identical procedure was applied to data recorded from all recording sites.

For each participant, the SSVEP data at each electrode associated with the 35 s epoch following the entrance to the next room was averaged across all of the 10 rooms.

For each participant, the mean phase of the SSVEP at each electrode evaluated over the first 5 s epoch following the entrance to the next room was set to zero before separately averaging the real and imaginary components of the SSVEP time-series across the 10 rooms. The SSVEP time series data at each electrode was then averaged across all participants to create the pooled SSVEP phase time-series data.

### Statistical Analysis

We used a permutation test to evaluate the hypothesis that the SSVEP phase at the FC5 site will exhibit a transient reduction at the time the exit door of the gallery room is viewed. We used a permutation test rather than a parametric test such as Student’s *t* for two main reasons. Firstly, parametric tests are based on the assumption that the test parameter is distributed normally. As the SSVEP phase data is not distributed normally, we used the permutation test which does not depend on any assumption of normality. In addition, the permutation test provides exact probabilities (Blair and Karniski, 1993). These features have led biomedical researchers to consider permutation tests as the preferred statistical methodology (Ludbrook and Dudley, 1998).

Our NULL hypothesis is that there is no statistical difference in SSVEP phase at the time the exit door is viewed compared to the mean SSVEP phase during the first 5.0 s of the trial. The mean of the first 5 s of the trial are used as the reference level as during this interval no paintings have been viewed in the current room. The mean SSVEP phase evaluated over the first 5.0 s of the trial is subtracted from all the SSVEP phase values in the 35 s epoch yielding an SSVEP phase difference time series for each electrode. To conduct the permutation test, a random subset of SSVEP phase difference time series are multiplied by −1 and a new average determined. This multiplication by −1 is equivalent to interchanging the SSVEP mean of the first 5 s and the SSVEP value at a given point in time and is consistent with the NULL hypothesis where it is assumed that the SSVEP phase is as likely to exceed the reference level as it is to be exceeded by the reference level. This process is repeated 100,000 times and the number of times where the permuted average of the SSVEP phase difference is equal to or less than the observed value at the 30 s point in time (the time of the appearance of the door) is determined. This number when divided by the number of permutations (100,000) yields the exact one-sided *p*-value of the observed SSVEP phase difference occurring under the NULL hypothesis. This process was repeated for all 20 recording sites.

As the hypothesis of this study is that the appearance of the exit door will be associated with a transient reduction in activity at electrode site FC5, we only consider the statistical significance of the activity on the appearance of the exit door at the 30 s point in time.

## Results

The SST based measures of activity during the 35 s epoch are illustrated in Figure 2 and the first appearance of the door at the 30 s mark is associated with a transient activity reduction that is most prominent at left frontal sites FC5 and C3.

Table 1 lists the exact probability of falsely rejecting the NULL hypothesis at the 30 s point in time for all electrode sites. While our hypothesis only refers to effects at electrode site FC5 we will extend our consideration beyond this single site and thus adjust the experiment wide p level to account for the 20 comparisons considered. After applying a Bonferroni correction for the 20 comparisons, only sites FC5 and C3 demonstrate an experiment wide effect significant at the corrected *p* < 0.01 level. Applying a less conservative criterion of an experiment wide effect significant at the corrected *p* < 0.05 level indicates a significant effect at the left parietal site P3. Statistically, the strongest effect was observed at FC5 and C3 where both are associated with a value of *p* = 2 × 10^–4^. Using the *r*_equivalent_ approach suggested by Rosenthal and Rubin (2003) to determine experimental effect size of this SSVEP reduction, we see that the effect size is *r* = 0.52, which is considered a large effect (Cohen, 1988).

**TABLE 1:** Exact probability of rejecting the NULL hypothesis that activity at the 20 recording sites is not less than the mean of the initial 5 s value.

**Electrode**	***p***	**Electrode**	***p***	**Electrode**	***p***
Fp1	0.0045			FP2	0.0270
F3	0.0062	Fz	0.0103	F4	0.0043
FC5	0.0002**			FC6	0.0783
T7	0.0134			T8	0.0840
C3	0.0002**	Cz	0.0178	C4	0.0385
P3	0.0011*	Pz	0.0198	P4	0.0542
PO7	0.0201			PO8	0.1972
O1	0.3129	Oz	0.1697	O2	0.6308

*One asterisk indicates that the effect is significant at the experiment wide *p* < 0.05 level after correcting for multiple comparisons, that is *p* < 0.05/20. The two asterisks indicate that the effect is significant at the experiment wide *p* < 0.01 or *p* < 0.01/20.*

In summary, we note that the strongest effects associated with the appearance of the door at the 30 s mark are restricted to the left frontal sites.

## Discussion

Our hypothesis was confirmed in that the appearance of the event boundary represented by the ‘virtual gallery door’ was associated with a robust transient drop in activity at FC5 the left frontal scalp site overlaying the ventro lateral prefrontal cortex (vlPFC). Given the recognized role of the vlPFC in long-term memory encoding (Buckner et al., 1999; Blumenfeld and Ranganath, 2007) and our previous work demonstrating a *Dm* effect for the SSVEP phase at FC5 the scalp site (Silberstein et al., 2000a), our current findings suggest that an event boundary triggers a transient reduction in long-term memory encoding. Such a reduction is consistent with the fMRI findings of Nakano et al. (2013), Nakano (2015) who interpreted their findings of blink related activity reduction in nodes of the dorsal and ventral attentional networks in terms of event boundaries causing a ‘transient disengagement from external perception and the engagement of internally oriented network,’ presumably such as the default mode network. Interestingly, Nakano et al. (2013) observed a blink related activity reduction at the right vlPFC while we observed the event boundary related reduction at FC5 located in the vicinity of the left vlPFC. We suggest two possible factors may have contributed to this difference. One could be the different events used to initiate data acquisition in our respective studies. We used explicit events (doorways) that are widely considered to comprise event boundaries (Radvansky and Copeland, 2006; Radvansky et al., 2011, 2015). By contrast, the Nakano et al. (2013), Nakano (2015) studies focused exclusively on blink related changes in brain activity. While event boundaries are associated with blinking, not all blinks are associated with event boundaries. For example, lapses or reductions in attention also cause increased blinking (McIntire et al., 2014). The possible contribution of factors other than event boundaries to the Nakano et al. (2013), Nakano (2015) right vlPFC findings is thus consistent with the right vlPFC comprising a node of the ventral attentional network (Vossel et al., 2014) and such attention effects are observed most consistently in the right hemisphere rather than the left (McIntire et al., 2014).

Another possible contributing factor is that participants in the Nakano studies were told that their recollection of the material viewed while in the scanner would be tested after the scan. By making the task one of memory recall, it is possible that this reduced the impact of event boundaries on long-term memory encoding and hence attenuated the event boundary related drops at the left vlPFC.

Our findings are also consistent with those of Ben-Yakov et al. (2014), Ben-Yakov and Henson (2018) who reported increased hippocampal activity immediately following the occurrence of event boundaries in a film viewed by participants. Increased activity in the hippocampus and other DMN nodes when perceiving an event boundary would result in transient decreased activity in task-oriented networks including the vlPFC (Buckner et al., 2008). This would in turn be associated with a transient reduction in attention and long term memory encoding. As the transient reduction in long-term memory encoding following an event boundary occurs in conjunction with increased hippocampal activity, this suggests that this transient reduction coincides with better encoding of the experience preceding the event boundary.

We observed a frontal left hemisphere bias in the strength of the event boundary related drop in SSVEP phase. This was the case even though the viewing material was primarily pictorial with no speech and text restricted to the painting labels. This left hemisphere bias is consistent with our earlier report on the SSVEP *Dm* effect (Silberstein et al., 2000a) as well as numerous studies pointing to a preferential role for the left vlPFC in the control of both encoding and retrieval of long-term memory (Badre and Wagner, 2007; Blumenfeld and Ranganath, 2007). Furthermore, our findings suggest that the left prefrontal and frontal cortex play an important role in not only encoding information into long-term memory but also in the segmentation of time extended events and the identification of high-level event boundaries. In summary, our findings point to the left frontal cortex playing an important role in interposing a transient drop in long-term memory encoding on the perception of an event boundary.

While our hypothesis is confirmed in that activity at the left frontal site FC5 showed a sharp drop at the event boundary, the findings of Ben-Yakov and Henson (2018) call into question the underlying mechanism we proposed for the event boundary effect. We proposed that the drop in vlPFC activity was driven by an increase in DMN and hippocampal activity at the event boundary. However, Ben-Yakov and Henson (2018) reported bilateral and approximately equal increases in hippocampal activity on encountering an event boundary. If the vlPFC drop was determined by increased ipsilateral hippocampal activity, then we might expect to see a drop in both left and right vlPFC activity or at scalp sites FC5 and FC6. We suggest that two possible explanations may account for the inconsistency between the neural mechanism proposed to account for the vlPFC event boundary effect and our observation of an overwhelmingly left hemisphere effect. One possibility is that the interaction between hippocampus and vlPFC may differ between left and right hemispheres. This would be consistent with the differing roles of the left and right vlPFC. The left vlPFC is known to play a crucial role in the control in both encoding and retrieval of information from long-term memory (Badre and Wagner, 2007). By contrast the right vlPFC is involved in non-memory processes such as response inhibition, etc. (Levy and Wagner, 2011).

Another possibility is suggested by a consideration of Event Segmentation Theory which proposes that an ‘event comprehension systems’ make predictions about upcoming events and an event boundary is identified when a prediction of upcoming events is in error (Swallow et al., 2009). Such prediction errors are associated with increased hippocampal theta activity suggesting increased encoding in long-term memory (Paller and Wagner, 2002; Garrido et al., 2015). A magnetoencephalography study of brain activity responses to a sequence of pictorial stimuli that violate expectations indicated that an EEG theta frequency mismatch signal is generated in a network comprising the hippocampus and ventro-medial prefrontal cortex (vmPFC) (Garrido et al., 2015). The authors report that it is the vmPFC that drives hippocampal activity when expectations are violated and that this effect is restricted to the left vmPFC and hippocampus. Such a left hemisphere bias is also consistent with our findings.

One possibility that must be considered is the extent to which our findings are due to visual sensory effects rather than the appearance of an event boundary. If sensory driven changes in visual attention were responsible for our observations, we would expect to see such changes reflected in activity at occipital sites (Silberstein et al., 1990). We consider this possibility unlikely for the following reason. While the appearance of the ‘door’ was associated with a robust SSVEP phase reduction at left frontal sites FC5 and C3, none of the occipital sites demonstrated statistically significant changes or differences with respect to the reference level at this time. However, while the SSVEP phase values at occipital sites are consistent with our suggestion that the effects at FC5 and C3 are unlikely to be triggered by visual sensory effects, our experimental design and methodology does not allow us to completely eliminate this possibility. For example, it may be possible that changes in activity at some components of the visual processing system in either the dorsal or ventral visual processing systems may account for our observations at FC5 and C3. One possibility suggested by a reviewer is that the Limited Capacity Model of Motivated Mediated Message Processing (LC4MP) may provide an alternative interpretation for our findings (Lang, 2006). Specifically, changes in the visual environment in moving from one gallery room to the next engages increased visual processing resources which in turn decreases performance in a ‘secondary task’ such as long-term memory encoding. One interesting approach suggested by the reviewer is a study where the visual environment remains unchanged while the event boundary is presented verbally, such as in the conclusion of a narrative or a verbal joke.

A point which we believe warrants comment is the relatively strong effect size of the observed event boundary related drop in SSVEP phase at FC5 (*r* = 0.52). We observed a comparably large effect size in our original report describing the *Dm* effect evaluated with the SSVEP (Silberstein et al., 2000a). While signal to noise considerations may play a role in contributing to the experimental effect size, we suggest that more fundamental aspects of neural information processing in the cortex may play an important role. Specifically, we suggest that the 13 Hz frequency used to elicit the SSVEP may be an important factor influencing the effect size. This is suggested in the light of recent findings concerning the neural mechanisms mediating sensory-driven or ‘bottom-up’ and cortical control or ‘top down’ information flows within the cortex. While these are briefly described here, readers seeking a more detailed description are referred to Silberstein et al. (2016). It is now recognized that both ‘bottom-up’ and ‘top-down’ intracortical communication processes are mediated by synchronous oscillations. Recent work examining the difference between the information flows reveals that they are mediated by oscillations in different frequency ranges. Specifically, bottom-up communication appears to be mediated predominantly by synchronous oscillations in the EEG gamma frequency range (40–80 Hz) while top-down communication is mediated predominantly synchronous oscillations in the EEG high alpha-low beta range (10–20 Hz) (Von Stein et al., 2000; Markov et al., 2014; Fries, 2015). Our choice of 13 Hz as the frequency used to elicit the SSVEP means that our responses (measurements, recorded findings) are likely to be driven predominantly by top-down processes that among other things would modulate long-term memory encoding. In other words, our SST methodology may be particularly well suited to measuring top-down processes and this may contribute to the strong effect size we have observed.

We have termed event boundary related transient drop in the SST based measure of activity at FC5 and C3 *Conceptual Closure*. We have frequently observed what we take to be Conceptual Closure when recording SST brain activity in naturalistic time extended stimuli such as television advertisements (Silberstein and Nield, 2008). While Conceptual Closure appears at obvious event boundaries such as the screen changing color or music ending, we have also observed it in more subtle indications of event boundaries such as two people standing to shake hands or a car driving into the distance. Consistent with our finding that long-term memory encoding at branding is associated with television advertising effectiveness (Silberstein and Nield, 2008) we have found that Conceptual Closure can have a significant negative impact of the commercial effectiveness of an advertisement if it coincides with the sole appearance of product branding or key message. This is typically the case where a television advertisement is structured as a narrative that describes the issue or problem to be addressed in the absence of an explicit or implicit representation of the brand or product in question. Frequently, the narrative concludes immediately before the appearance of the product or brand and thus the narrative conclusion is processed by the brain as an event boundary triggering Conceptual Closure at the time the product or brand first appears in the advertisement. We have found this situation is more likely to be associated with consumers associating a competing brand with the advertisement.

### Illustrative Case Study

The following case study is provided to illustrate the manner in which *Conceptual Closure* can be managed so as to enhance an advertisement’s commercial effectiveness. It should be stressed that the following case study was undertaken as a standard commercial study for a client as advertiser. It is primarily presented here to illustrate how findings concerning *Conceptual Closure* are dealt with in a commercially relevant manner.

Neuro-Insight Pty Ltd. was commissioned to conduct a two-part advertising research study examining an advertising campaign by the client, NAB Ltd. a major Australian financial services provider for its superannuation fund, MLC. The design of the advertising campaign, named ‘Save Retirement’ was to encourage likely clients to seek retirement related financial advice from MLC. The campaign was launched in early 2014 and had been on air for approximately 3 months with independent tracking indicating the campaign was suffering from poor brand linkage in that an unacceptably high proportion of viewers were inclined to identify a competing brand as the advertiser.

In the first part of the study, Neuro-Insight evaluated the effectiveness of the original 30 s television advertisement on the basis of SST measures of long-term memory encoding (scalp site FC5) at the point of branding in the final 5 s of the advertisement. Recommendations on how to address identified deficiencies were made and implemented by the creative agency with a re-edited 30 s advertisement broadcast in the last quarter of 2014. The re-edited advertisement can be viewed at https://www.youtube.com/watch?v=ZbzKcTaOlXg. The original advertisement was on air for 3 months and the re-edited advertisement for a following 2 months. An independent market research firm was commissioned by the client to track the performance of both the original and revised advertisement. As this was a standard commercial study conducted by Neuro-Insight it was undertaken in accordance with the ESOMAR *International Code on Market, Opinion and Social Research and Data Analytics* and the Neuromarketing Science and Business Association (NMSBA) *Code of Ethics*. It should also be noted that the data recording procedures and equipment used in this case study are identical to those used in the preceding study and approved by the Swinburne University Human Research Ethics Committee as application SUHREC2014/029.

Studies of the original and re-edited advertisement were both conducted in Melbourne at the Neuro-Insight offices. Fifty participants (25 females) with a mean age of 54 years (range 45–64 years) participated in the first study undertaken in July 2014. Another group of 50 participants (25 males) with a mean age of 52 years (range 45–61 years) took part in the subsequent study undertaken in September 2014. Participants in both studies were recruited through a recruitment agency and had to satisfy a range of criteria such as age, employment status and financial decision-making responsibility as specified by the client. Nine participants in the first study (six females) also participated in the subsequent study.

In both studies, participants viewed the client television advertisement in the context of a typical television viewing experience. The advertisement was viewed as one of a group of four advertisements in an advertising break occurring in a typical prime time television program.

A total of three groups of advertisements or advertising ‘breaks’ were present in both studies with the presentation of the client advertisement counterbalanced in either the first or second advertisement break.

The advertisement was viewed in one of two groups of advertisements or advertising breaks. In both cases, the client advertisement occurred as the second advertisement in the break and was preceded by a different advertisement in each break. The order in which the advertisement was viewed was evenly rotated across the entire sample. All details of the procedure, data acquisition and analysis are the same as those described in the multi room gallery study described above.

Both versions of the advertisement featured a young boy and his grandfather visiting a futuristic ‘museum’ where a display features a happy older couple going for a drive in the country in their sports car, and by inference enjoying their retirement. As they look at the display, the young boy asks ‘what are they doing’ and the grandfather wistfully replies ‘they are in retirement’ and in retirement ‘they can do anything they want.’ In the original advertisement, a voice-over then suggests people speak to their financial advisor and subsequently the boy pulls his grandfather away and they both leave the display.

Analysis of the original advertisement indicated two transient reductions in long-term memory encoding in the final 7 s of the advertisement. The first at the 22.5 s mark coincides with the end of the story narrative and a verbal call to action. The second at the 24 s mark coincided with the boy pulling his grandfather from the ‘retirement display.’ We consider both drops in long-term memory encoding to be examples of *Conceptual Closure* as they occurred at clear event boundaries in the advertisement. The likely impact of Conceptual Closure on the effectiveness of the advertisement is indicated by the level of long-term memory encoding during branding during the final 3.5 s. The brand is only featured in the last 3.5 s of the original advertisement (final branding) and during this period, long-term memory encoding is low and in the lowest 8% of our database of financial services advertisements.

**FIGURE 3:**
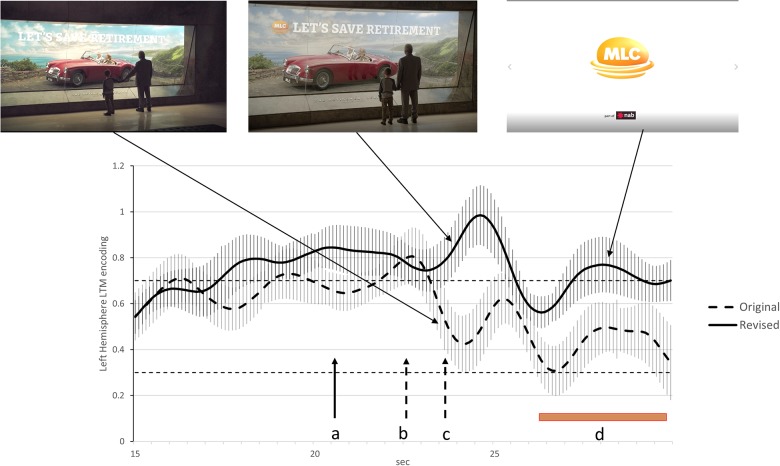
Activity at FC5 indicating levels of long-term memory encoding for the last 15 s of the original and re-edited advertisements. The dashed line illustrates long-term memory encoding for the original advertisement while the solid line is the equivalent finding for the re-edited advertisement. The 84% confidence interval are included for both time series and when these do not overlap, the statistical significance of the difference between time series corresponds to *p* < 0.05 (Austin and Hux, 2002). The points in time where the narrative ends and the call to action starts in the original advertisement is indicated by the dashed arrows labeled ‘b’ while the dashed arrow labeled ‘c’ indicates when the child starts pulling his grandfather from the display cabinet. The solid arrow labeled ‘a’ indicates the point in time where the narrative end and the call to action starts in the re-edited advertisement. The brown bar labeled ‘d’ indicates the duration of the logo on the screen and final branding. The removal of the segment where the boy pulls his grandfather away from the cabinet appears to reduce the impact of Conceptual Closure and lead to higher long-term memory encoding during the final 3 s of the advertisement.

The main changes recommended for the revised advertisement were aimed at reducing the impact of *Conceptual Closure* at the time of branding. These included introducing the brand verbally and visually earlier to suggest a continuation of the original narrative while the grandfather and boy remain at the display. Furthermore, the scene triggering Conceptual Closure was edited out so that the boy and grandfather remained stationary throughout the advertisement. To accommodate these changes, the end of the narrative and call to action occurred 2 s earlier. The impact of these changes on long-term memory encoding at the times of branding was significant in that the level of long-term memory encoding at time of the newly branded scene peaked at the 96th percentile of our database of financial services advertisements while the final branding final visual branding was now at the 72nd percentile of our database of financial services advertisements. These differences in long-term memory encoding at the time of branding were significant at the *p* < 0.05 level and this is explained in more detail in the Figure 3 legend.

During the 3 months period that the original advertisement was aired and the following 1 month where the re-edited advertisement was being broadcast, an independent market research firm conducted advertising tracking studies for the client. It was found that in the 3-month period when the original advertisement was being aired, the proportion of viewers who had seen the advertisement and correctly identify the brand (brand linkage) was considered low by the market research firm conducting the tracking study. However, in the first month that the re-edited advertisement was being broadcast, we were advised by the client that the market research firm conducting the tracking study reported that brand linkage had more than doubled, increasing by 120%. While the tracking findings are consistent with the SST indications of improved advertising effectiveness, we cannot exclude the possibility that other factors external to those determined by the client may have also contributed. However, the client is unaware of any such external factors that may have contributed to the advertisement’s improved performance.

### Limitations and Future Research

We acknowledge that one of the limitations of this study is that the experimental design does not enable us to include behavioral data to confirm the drop in long-term memory encoding on the appearance of the door. We have relied on extensive evidence from other laboratories on the role of the left ventrolateral prefrontal cortex and long-term memory encoding as well as our previous findings demonstrating the link between activity at FC5 long-term memory encoding to infer a drop in long-term memory encoding on the appearance of the event boundary. Nevertheless, the incorporation of a behavioral test of long-term memory encoding at the event boundary is a recommendation for future studies.

Another limitation concerns the limited amount of information that could be included in the illustrative case study concerning the effectiveness of the first and then the revised advertising phases. As this was a commercial study, the client has the final say on what, or if any commercially sensitive information concerning the study may be published. We are grateful to MLC for their permission to include the percentage increase in the brand linkage and advise that brand linkage data for the first and revised advertising phases has not been made available for publication.

## Conclusion

Event boundaries can trigger a brief drop in long-term memory encoding or *Conceptual Closure*. While Conceptual Closure, in general may not compromise advertising effectiveness, it may do so if it occurs at the time that a key message or branding is presented. In the case study, we show how minor changes to a finished advertisement can modify the impact of Conceptual Closure and in turn significantly enhance advertising effectiveness.

## Data Availability Statement

The datasets generated for this study are available on request to the corresponding author.

## Ethics Statement

The studies involving human participants were reviewed and approved by the Swinburne University Human Research Ethics Committee as application SUHREC2014/029. The patients/participants provided their written informed consent to participate in this study.

## Author Contributions

RS and GN conceived the project. SS and GN supervised development of video first person journey. RS and SS contributed to the experimental design. SS supervised the project. RS developed statistical analysis software for the study and analyzed the data.

## Conflict of Interest

RS, SS, and GN are employed by Neuro-Insight Pty Ltd. and have a financial interest in Neuro-Insight Pty Ltd.
